# “I aspire to look and feel healthy like the posts convey”: engagement with fitness inspiration on social media and perceptions of its influence on health and wellbeing

**DOI:** 10.1186/s12889-018-5930-7

**Published:** 2018-08-10

**Authors:** Michelle Raggatt, Cassandra J. C. Wright, Elise Carrotte, Rebecca Jenkinson, Kate Mulgrew, Ivanka Prichard, Megan S. C. Lim

**Affiliations:** 1 Burnet Institute, 85 Commercial Rd, Melbourne, 3004 Australia; 2 School of Population Health and Preventive Medicine, Monash University, Melbourne, Australia; 3 Australian Institute of Family Studies, Melbourne, Australia; 4 School of Social Sciences, University of the Sunshine Coast, Maroochydore, Australia; 5 Health & Exercise Sciences, College of Nursing and Health Sciences, Flinders University, Adelaide, Australia; 6 Melbourne School of Population and Global Health, The University of Melbourne, Melbourne, Australia

**Keywords:** Social media, Fitspiration, Body image

## Abstract

**Background:**

Fitspiration is a popular social media trend containing images, quotes and advice related to exercise and healthy eating. This study aimed to 1) describe the types of fitspiration content that users access and how they engage with content, 2) investigate the disordered eating and exercise behaviours and psychological distress of individuals who access fitspiration, and 3) understand the perceived influence of fitspiration on health and wellbeing.

**Methods:**

Participants who access fitspiration content were recruited via social media to complete a cross-sectional online survey. Participants’ psychological distress was measured using the Kessler 10 Psychological Distress Scale (K10); disordered eating behaviours using the Eating Attitudes Test-26 (EAT-26); and compulsive exercise behaviours using the Exercise Addiction Inventory (EAI). Participants also answered a series of open-ended questions about their experiences with fitspiration. A descriptive statistical analysis was conducted for quantitative data. Responses to open-ended questions were analysed for key themes using an iterative process of open, axial and thematic coding.

**Results:**

Participants (*N* = 180, 151 female, median age 23.0 years (IQR 19.0, 28.5)) most commonly accessed content posted by personal trainers and athletes (59.4%), posts tagged with the ‘fitspiration’ hashtag (53.9%) and posted by ‘everyday’ people (53.3%). Overall, 17.7% of participants were classified as high risk for an eating disorder, 17.4% reported very high levels of psychological distress, and 10.3% were at risk of addictive exercise behaviours. Participants described both positive and negative influences of engaging with fitspiration content. The influence on their health beliefs and behaviours was explained through four key themes: 1) Setting the ‘healthy ideal’, 2) Failure to achieve the ‘ideal’, 3) Being part of a community, and 4) Access to reliable health information.

**Conclusions:**

Many participants reported benefits of fitspiration content including increased social support and access to health information. However, participants also reported that fitspiration content could negatively influence their wellbeing and perception of healthy goals. Content posted by relatable individuals or qualified experts was perceived as most trustworthy. Future research is needed to determine the individual and content-related factors associated with negative and positive fitspiration experiences.

## Background

Increasingly, people look to social media to connect with each other, create expressions of self-identity, and seek information about health [1–3]. ‘Fitness inspiration’, often abbreviated to ‘fitspiration’ or ‘fitspo’, is a popular health trend on social media where individuals post or view images, quotes and advice about fitness and nutrition [4]. Some social media users who follow the fitspiration trend also engage in discussions that shape an online ‘fitness culture’ including expressing views around a ‘healthy’ appearance and ‘correct’ dieting and exercise behaviours [5]. Fitspiration content is generated and shared on major social media platforms such as Instagram, Facebook and Tumblr with ‘friends’, ‘followers’, or the general public [6]. ‘Hashtags (#)’ accompanying content enable it to be followed by other social media users. To illustrate its popularity, a search (October 9, 2017) of ‘#fitspo’ on Instagram returned over 48 million posts [7].

Despite an ostensible focus on healthy lifestyle behaviours, content analyses have revealed that fitspiration content commonly portrays several harmful themes [6, 8, 9]. For example, fitspiration content contains objectifying images that depict an idealised thin-athletic female body type and hypermuscular male body type [6, 8, 9]. Researchers have posited that while the shift from a focus on thinness to fitness may outwardly seem positive, the healthy looking ideal is still underpinned by aesthetic perfection [5]. In order to achieve this athletic-ideal body, individuals may be required to practice greater dietary restriction and engage in high intensity exercise regimes [10]. Content analyses also demonstrate that fitspiration content depicts themes related to restrictive eating and exercise practices [6, 8]. A common focus of fitness inspiration content is on exercising for appearance reasons [9], which has been associated with more negative body image [11, 12]. Messages on fitness inspiration pages such as ‘clean eating’ (eating foods perceived to be healthy and unprocessed) and guilt for eating ‘unhealthy’ foods may encourage people to diet or restrict certain food groups [9].

Experimental studies also suggest that acute exposure to fitspiration images can increase short-term body dissatisfaction and negative mood among female undergraduate students [4, 13, 14]. This is concerning as body dissatisfaction is a significant risk factor for eating disturbances including anorexia and bulimia nervosa [15, 16]. In addition, a study conducted with female participants who posted fitspiration on Instagram found that these participants reported more disordered eating and compulsive exercise behaviours compared to participants who posted about travel [17]. A survey of social media users aged 15–29 years in Australia also found that ‘liking’ or ‘following’ diet and fitness content, including fitspiration, on social media was associated with greater odds of self-reported history of an eating disorder and misuse of detox products or diet pills [18]. From a sociological perspective, fitspiration has been described as entrenching dominant discourses on health and the ideal body [5]. Despite the online interactive platform providing a safe space for users to challenge current orthodox ideals surrounding thinness which are presented by traditional media, fitspiration users internalised these ‘healthy’ ideals as truths [5]. Further, individuals were held morally accountable for regulating their bodies by adhering to normative health choices and behaviours [5].

While previous research highlights the potential harms of fitspiration, to date, content analyses have had a narrow scope, focussing on posts and websites explicitly identified as fitspiration either through hashtags linked to the posts (e.g., Tiggemann and Zaccardo [4]) or using a keyword search engine for websites (e.g., Boepple, Ata, Rum and Thompson [9]). Further, in the study by Holland and Tiggemann [17], researchers recruited only female participants who posted on social media with one hashtag (#fitspo). Less is known about other types of fitspiration content aiming to motivate users to engage in healthy eating and exercise, or which types of content are the most commonly viewed. Thus, in this paper we offer a broadened definition of fitspiration as a category of social media content including images posted to social media with the hashtags (#) ‘#fitspiration’ or ‘#fitspo’; as well as profile pages or blogs by personal trainers, fitness models or body builders; and content related to fitness challenges, diets, and health cleanses.

Research that specifically investigates individuals who post or follow fitspiration content is also limited. Experimental studies investigating the effects of fitspiration content recruited female undergraduate students regardless of whether they choose to engage in fitspiration [4, 13]. The study by Carrotte, Vella and Lim [18] included social media users who reported ‘liking’ or ‘following’ fitspiration; however, the survey did not use validated measures to capture information about body image or disordered eating and exercise behaviours. The study by Holland and Tiggemann [17] recruited individuals who posted fitspiration content; however, did not include individuals who only view content. Individuals who view, but do not post, fitspiration content are also exposed to the potentially harmful imagery and themes and are also likely to experience the negative effects associated with fitspiration content. Therefore, the current study aims to explore the characteristics and perceptions of individuals who post as well as view fitspiration content.

Finally, given few studies have investigated people who choose to follow fitspiration content, there is little evidence about how individuals perceive its impact on their health and wellbeing, including both the positive and negative effects. Understanding the perceptions of individuals who access fitspiration content will also enable a greater understanding of how fitspiration content shapes health beliefs and attitudes, which are theorised as important predictors of health behaviours [19].

Given these gaps in the current literature, this study aimed to answer the following research questions: 1) What are the characteristics of individuals who choose to engage with fitspiration content including psychological distress, and risk of disordered eating and exercise behaviours?; 2) What types of fitspiration content do people access and how do they engage with this content?; and 3) What is the perceived influence of fitspiration content?

## Methods

### Study design

This study employed a cross-sectional online survey consisting of closed-ended questions to capture quantitative data and open-ended questions to capture qualitative data.

### Participants

Participants were recruited through advertisements on Facebook and Instagram during a six-week period in June and July, 2016. Eligible participants were aged 16 years or older, reported engaging with at least one type of fitspiration content on social media, and lived in Australia. Advertisements were targeted to reach individuals whose profiles met the eligibility criteria and were also posted on Facebook group pages related to health and fitness where the researcher had gained approval from the group’s administrator. In addition to the targeted advertising strategy, participants also indicated their eligibility prior to the survey and were excluded from completing the survey if they gave an answer that was discordant with the eligibility criteria. Clicking on the advertisements directed participants to the online survey. There were 813 clicks from social media advertisements to the survey webpage, indicating a 22% (180/813) conversion rate of those who clicked on the link and completed the survey. No incentive or reimbursement was offered for participation.

### Data collection

The survey was pre-tested by the primary researcher’s colleagues, and feedback was sought to improve clarity of questions and functionality of survey administration. Total time to complete the survey (including collection of quantitative data) was approximately 30 min. A secure, web-based application, Research Electronic Data Capture (REDCap) [20], was used to collect and manage participant data. The following domains were included in the survey:

#### Demographic characteristics

Demographic characteristics were collected including age, gender, sexual identity, highest level of education completed, recreational spending money per week, and whether participants lived with their parents, or had any children. Body mass index (BMI) was calculated from participants’ self-reported height and weight. Area of residence, defined as major city or regional, was determined using participants’ self-reported postcode.

#### Psychological distress

Psychological distress was measured using the Kessler 10 Psychological Distress Scale (K10) [21, 22]. The scale consists of 10 items relating to feelings of fatigue, nervousness, hopelessness, restlessness, depression and worthlessness during the past 4 weeks [22]. Each item is scored on a five point scale ranging from 1-‘none of the time’ to 5-‘all of the time’, with scores of 30–50 indicating ‘very high’ psychological distress, 22–29 ‘high’, 16–21 ‘moderate’, and 10–15 ‘low’ [23]. Previous research has shown the K10 scale to have good validity and reliability [22, 24]. In the current sample, internal consistency was excellent (Cronbach’s α = 0.93).

#### Disordered eating behaviours

Disordered eating behaviours were measured using the Eating Attitudes Test-26 (EAT-26), a 26-item scale that measures attitudes towards food and eating (e.g. ‘I find myself preoccupied with food’) [25]. Participants indicated how often each item applies to them using a 6-point scale (0-‘never’, ‘rarely’ or ‘sometimes’, 1-‘often’, 2-‘usually’, 3-‘always’). A score of 20 or more indicates risk of an eating disorder. The EAT-26 has been found to have acceptable to excellent internal consistency and good validity in past research [25, 26]. The scale demonstrated good internal consistency in the present sample (Cronbach’s α = 0.89).

#### Compulsive exercise behaviours

Compulsive exercise behaviours were measured by the Exercise Addiction Inventory (EAI), a 6-item scale which measures risk of addictive exercise behaviours (e.g. ‘If I have to miss an exercise session I feel moody and irritable’) [27]. Items in the EAI are scored on a 5-point scale from 1-‘strongly disagree’ to 5-‘strongly agree’. The total score is categorised into ‘asymptomatic’ (scores 0–12), ‘symptomatic’ (13–23), and ‘at-risk’ of exercise addiction (24 or greater) [28]. Previous research has found good validity and internal consistency [27], while in the current sample, the EAI had acceptable internal consistency (Cronbach’s α = 0.69).

#### Fitspiration content

Participants indicated which types of fitspiration content they access on any social media platform by selecting from a checkbox list with the following types of content: ‘fitspiration’ posts; weight loss or body transformation motivation; personal trainers or athletes, celebrities and models (for the purpose of weight loss or fitness motivation); body building or strength training; ‘clean eating’; diets; fitness challenges; and detoxes/cleanses. Participants could select multiple responses. We also asked if they accessed ‘thinspiration’, another social media trend promoting a thin body type, extreme weight loss and advice for engaging in disordered eating behaviours content, as this is known to have negative effects for body perceptions and disordered eating behaviours [29]. We were interested in how many participants accessed both types of content as fitspiration is often credited as a healthier reaction to the thinspiration trend [30]. However, participants who only viewed thinspiration, not fitspiration, were not included in the current study.

#### Fitspiration engagement

Participants indicated how they engage with fitspiration content by selecting from a checkbox list of engagement behaviours (Table 3). Engagement behaviours were subsequently categorised into two groups to determine the level of engagement with fitspiration: 1) activities that involve actively contributing and sharing content (e.g., ‘Participate in discussions’), and 2) activities that involve more passive engagement through observing content (e.g., ‘Scroll through posts or images’) (Table 3). Previous studies investigating social media have used self-reported estimates of frequency and duration of exposure to social media content; however, such estimates can be heavily affected by recall bias and do not capture the different ways that people can interact with social media content or their level of involvement [31]. Instead, researchers and marketing professionals have suggested classifying social media users based on their behavioural engagement with content and theorise that those who participate actively with social media content may be more likely to make behavioural changes [32, 33].

#### Motives for accessing fitspiration content

We captured participants’ reasons for accessing fitspiration from a predefined checkbox list that included reasons related to improving health and wellbeing, diet, appearance, muscular strength, body shape and size, and because their friends like it (Table 4). Participants could select multiple reasons.

#### Perceived influence of fitspiration on health and wellbeing

The online survey contained three open-ended questions (Table 1). Questions were designed to elicit both positive and negative experiences with fitspiration. Open-ended questions were asked before specific questions relating to health outcomes and behaviours to maximise the emergence of new ideas and minimise bias due to perceived knowledge of the researcher’s intent and the subsequent questions about body image, mental health and exercise behaviours.

**Table 1:** Open-ended questions

1. What do you like the most about this content? 2. Does this content inspire you to exercise/eat healthily? Why? 3. Do you feel that viewing this content has influenced your health and wellbeing behaviour (e.g. changing your exercise or diet, influencing your mental health)? Please explain the ways that it has or has not? (This may be positive and/or negative)	

### Data analysis

Quantitative data analysis was performed using Stata version 13 [34]. We used descriptive statistics to determine the percentage of participants who accessed each type of fitspiration content and the way they engaged with it. Descriptive statistics are also provided for BMI, K10, EAI, EAT-26 and socio-demographic characteristics. Qualitative data were analysed using NVivo 11 Software [35]. A coding framework was developed after reading all responses, and was refined throughout an iterative process of open, axial, and thematic coding [36]. Open coding identified the discrete categories capturing perspectives, health behaviours and beliefs, and effects of fitspiration. Axial coding focused on identifying themes relating to similar meanings and ideas, relationships between participants’ responses and comparisons to the open codes. Thematic coding identified overarching themes that emerged from the conceptual links between open and axial codes. The coding and conceptual links were discussed amongst the research team to ensure consistency and to resolve any discrepancies. Illustrative quotes were copied verbatim with ‘sic’ used to denote words that appeared erroneous such as a typing mistake. To maintain anonymity, only individuals’ gender and age are reported.

## Results

### Sample characteristics

The overall sample included 180 participants. The majority of participants were female (*n* = 151, 84%), median age was 23.0 years (IQR 19.0, 28.5) and median BMI was 24.5 kg/m^2^ (IQR 21.6, 26.8) (Table 2). According to the EAT-26 scale, 17.7% (*n* = 21) of participants were classified as at high risk of an eating disorder. K10 scores indicative of psychological distress were highly prevalent with 25.4% (*n* = 35) of participants having ‘high’ and 17.4% (*n* = 24) having ‘very high’ psychological distress. From the EAI scale, 10.3% (n = 15) of participants were at risk of addictive exercise behaviours.

**Table 2:** Participant characteristics

Variable	Participants *N* = 180	Missing n (%)
Age (years), n (%)		0 (0)
16–24	100 (55.6)	
25+	80 (44.4)	
Gender, n (%)		0 (0)
Female	151 (83.9)	
Male	27 (15.0)	
Other^a^	2 (1.1)	
Sexual identity, n (%)		3 (1.7)
Heterosexual	147 (83.0)	
Not heterosexual	30 (16.9)	
Area of residence in Australia, n (%)		9 (5.0)
Major city	143 (83.6)	
Regional	28 (16.4)	
Education, n (%)		0 (0)
Post high school	137 (76.1)	
No post-high school	43 (23.9)	
Have a child/children, n (%)		1 (0.6)
No	149 (83.2)	
Yes	30 (16.8)	
Live with parents, n (%)		1 (0.6)
No	95 (53.1)	
Yes	84 (46.9)	
Recreational spending money, per week n (%)		5 (2.8)
> $80	70 (40.0)	
< $80	105 (60.0)	
Body mass index, median (IQR)	24.5 (21.6, 26.8)	5 (2.8)
K10^b^, median (IQR)	20 (14, 27)	42 (23.3)
Exercise addiction inventory, mean (SD)	18 (4.3)	34 (18.9)
EAT-26^c^, median (IQR)	7 (4, 15)	61 (33.9)

^a^Other gender (*n* = 2: male and female = 1, non-binary/genderqueer = 1), ^b^K10: Kessler 10 Psychological Distress Scale, ^c^EAT-26: Eating Attitudes Test-26 scale

### Fitspiration content and engagement

Table 3 summarises the types of content accessed by participants and the ways that they engaged with fitspiration content. The most popular types of content accessed by participants were content posted by personal trainers and athletes (*n* = 107, 59.4%), followed by posts tagged with the ‘fitspiration’ hashtag (*n* = 97, 53.9%), posted by ‘everyday’ people (*n* = 96, 53.3%), and ‘clean eating’ (*n* = 93, 51.7%). The majority of participants (*n* = 117, 65.0%) only reported passive engagement activities and 35% (*n* = 63) reported any activities involving contributing fitspiration content.

**Table 3:** Fitspiration content accessed and engagement activities

	Participants, n (%) *N* = 180
**Type of fitspiration content**	
Personal trainers/athletes	107 (59.4)
Posts tagged with fitspiration or fitspo	97 (53.9)
Everyday people	96 (53.3)
Clean eating	93 (51.7)
Fitness challenges	80 (44.4)
Weight loss/body transformation	76 (42.2)
Body building/strength training	66 (36.7)
Diets	48 (26.7)
Celebrities	45 (25.0)
Models	38 (21.1)
Dieticians	25 (13.9)
Detox cleanses	23 (12.8)
Thinspiration	14 (7.8)
Other	9 (5.0)
Engagement activity	
Passive observing activities:	
Appears on newsfeed	164 (91.1)
Like/follow	110 (61.1)
Scroll through posts or images	101 (56.1)
Friends like/post/share	83 (46.1)
Visit related websites/pages/ profiles	77 (42.8)
Search hashtags	47 (26.1)
Other	1 (0.6)
Active contributing activities:	
Tag friends in posts	28 (15.6)
Comment on posts	27 (15.0)
Post content	24 (13.3)
Share with friends	23 (12.8)
Participate in discussions	22 (12.2)
Maintain/moderate a page	7 (3.9)

Note: Participants could select multiple options

### Reasons for accessing fitspiration content

The majority of participants (*n* = 159, 90.3%) reported that fitspiration content inspired them to exercise or eat healthy. Furthermore, the most common reasons for accessing fitspiration content related to health and wellbeing such as “To inspire me to exercise to improve my health or wellbeing,” selected by 73.9% (*n* = 133) of participants (Table 4). However, reasons related to weight loss and appearance were also common with 53.9% (*n* = 97) of participants selecting “To inspire me to exercise or diet to lose weight” and 42.2% (*n* = 76) selecting “To inspire me to improve my appearance.”

**Table 4:** Reasons why participants access fitspiration content on social media

Reason	n (%) *N* = 180
To inspire me to exercise to improve my health or wellbeing	133 (73.9)
To inspire me to eat healthy food	115 (63.9)
To learn more about health and wellbeing	114 (63.3)
To inspire me to improve my body shape, tone, or size	103 (57.2)
To inspire me to exercise to gain muscle/become stronger	102 (56.7)
To inspire me to exercise or diet to lose weight	97 (53.9)
To inspire me to improve my appearance	76 (42.2)
My friends view it/like it	24 (13.3)

Note: Participants could select multiple options

### Perceived influence of fitspiration

Responses from 155 participants who completed the open-ended questions were analysed. The number of words provided per participant ranged from two to 227 words, with a median of 36 words. Most participants perceived fitspiration had influenced their health through thinking about, changing, or maintaining their diet and exercise behaviours. Four key themes emerged regarding how fitspiration content had influenced their health beliefs and behaviours: 1) Setting the ‘healthy ideal’, 2) Failure to achieve the ‘ideal’, 3) Being part of a community, and 4) Access to reliable health information.

#### Setting the ‘healthy ideal’

Approximately half of our participants saw fitspiration images as portraying an ideal representation of health and fitness that they wanted to strive towards, for example, *“I use the images for goal setting i.e. I could look like her”* (Female, 18). They described using the ideal image as their primary goal for increasing their exercise and healthy eating. Subsequently, their goal image became the benchmark from which participants compared themselves and measured their success. These participants were determined to work hard towards their ideal goal until they reached it. They did not discriminate between themselves and others’ potential for a certain appearance or body type, but felt that by applying enough determination, effort, discipline and hard work to their goal, it could be achieved:

*“It’s helped [me] work harder and shows that when effort and determination is put in you can always reach your goal.”* (Female, 16).

Furthermore, one participant described a lack of dedication and commitment to the ideal as a sign of weakness and disregard for health:

*“I don’t think ‘fat acceptance’ should be a thing. It’s just laziness. By looking at pictures of fit girls and learning about good food and workout routines, it keeps your mind on the right track.”* (Female, 27).

For female participants, entwined in the notion of an ideal goal was the perception that achieving this goal equated to success and would bring happiness:

*“…I want to look like them and be able to do the strength/flexibility feats they can do (Pole dance/yoga/handstands). I think I will be happier if I do.”* (Female, 25).

The perceived ideal appearance for female participants was to look strong, fit and toned. The fitspiration trend had contributed to a shift in the ideal body image from thin to strong, and had reduced stigma around weight training and muscle building for females:

*“Women are taught cardio is the way and weights will make you ‘too big’ but at the moment there are so many strong insta*[sic] *famous ladies who are killin*[sic] *it.” (Female, 23*).

As the trend in cultural body ideals had changed, so too had their personal body goal. Participants praised the shift towards strength and fitness as a healthier trend and also credited it with improving their sense of a positive body image and focussing more on health:

*“This content has motivated me to join the gym and has also changed my ideal body goal from thin/extreme weight loss to strong/healthy, therefore having a healthier body image.” (Female, 18).*

It became apparent that, for about a fifth of participants, their idea of being healthy had often become conflated with looking healthy. Participants suggested that attaining the ideal appearance, as portrayed by fitspiration images, equated to optimal health, fitness and strength. For example, *“I aspire to look and feel healthy like the posts convey” (Female, 18).*

Among the relatively small number of male participants, about a third similarly emphasised slimness and strength as their ideal body goal, however, responses from males were more likely to specifically emphasise muscular “gains,” size and definition. One participant explained his current routine of increasing muscle mass and tone, *“Through reading and studying what certain athletes have done (how they eat and train) ... And because of this I went from 130kgs to 78kgs. Now currently in the routine of putting muscle on in the off season and toning in season.” (Male, 27).*

#### Failure to achieve the ‘ideal’

Approximately one-quarter of responses conveyed an underlying sense of feeling inadequate in terms of their appearance, fitness level and overall sense of worth as many female participants cited a desire to be “better”, “stronger” or “fitter” than they currently were. A small number of female participants assigned themselves blame and reported failing to reach their goal when they compared their progress and appearance to the fitspiration trend. Failure to meet their perceived ideal goal made them question their worth as a person saying that, *“…it makes me upset that I don’t feel I look good enough to start with” (Female, 20)*.

Another participant spoke of the pressure to look like the ideal and resulting sense of inadequacy as negatively affecting her recovery from an eating disorder: *“It has definitely impacted my mental health and has probably slowed recovery from my eating disorder. It can cause anxiety and hopelessness to know that I don’t and will never look like ‘fitspiration’ people.” (Female, 19).*

Only one male participant expressed body-image related concerns for males who may compare themselves to images depicting hyper-muscularity “*when they see photos of a male claiming to be natural but using steroids.”* (Male, 22).

In contrast, a small number of participants demonstrated a critical awareness surrounding the potential for fitspiration to negatively affect body image by setting unrealistic goals. These participants acknowledged that an ideal body was often unattainable and depended on genetic factors or “unrealistic” extreme regimes. They reported that, *“… they look that way mostly because of their natural body type and that no amount to*[sic] *exercise or healthy eating is guaranteed to make someone else appear just as good.”* (Female, 19). Furthermore, a few participants were cynical towards the idea of appearance-related goals. These participants valued content that emphasised the skilful and functional aspects of fitness and they preferred to set performance-related goals. For example, *“If it’s done correctly and it focuses on building strength or improving stamina, it can be motivating. A lot of us know we’ll never be VS* [Victoria Secret] *angels so I think we’re moving away from wanting to look like that and focusing more on improving ourselves to what our body can achieve”* (Female, 16).

#### Being part of a community

Participants also perceived fitspiration as an online community for like-minded people to share their interest in health and fitness. This community offered participants a sense of support and sharing in each other’s health and fitness ‘journey’. Participants felt a sense of accountability through a shared commitment to strive towards their health and fitness goals. Rather than having an individual goal they saw improving health and fitness as a shared goal. Participants enjoyed *“feeling a part of the larger fitness community and sharing it with friends”* (Male, 24) and stated that, *“We’re all working towards something amazing”* (Female, 27).

A small number of participants felt that having an online fitness community was beneficial because they did not have access to such a community offline. This related to the advantages of social media: to increase accessibility and connect people with similar interests. For example, *“… it gives you the accountability and support without having to have a group of people around you, can be very motivating”* (Female, 25).

While some participants stated that they followed their ‘role models’ or ‘idols’, more commonly reported by about a third of participants was feeling inspired by looking at the success stories and progress of ‘everyday’, ‘normal’ people posting fitspiration content. This inspiration was predominantly due to being able to relate to the person posting; they were perceived as a person who faced similar challenges and barriers to getting healthy. Therefore, they felt that the results of others were also achievable for themselves:

*“It is inspiring to see normal people doing what I wish to achieve”* (Male, 16).

*“If they can do it then there is no reason why I can’t.”* (Female, 25).

There was also a greater sense of trust about those who were ‘everyday’ people because participants felt that they give a more honest account of their difficulties as opposed to a paid fitness model or actress who could give a superficial, glorified view of becoming fit, be sponsored by a company, or understate the challenges of changing their lifestyle. These participants were selective about the content they followed and deliberately avoided content perceived to be ‘superficial’ or ‘fake’:

*“I have to make sure that the pages I follow are realistic. I don’t follow any fashion models or people that will make me feel shit about myself. I like to follow people who are honest about how hard it can be to lose weight and to stay healthy.”* (Female, 25).

#### Access to reliable health information

About half of participants frequently commented that they enjoyed learning about health and fitness through their online communities. Being a part of the fitspiration trend reportedly gave participants greater access to healthy recipes, exercise ideas and knowledge about fitness and nutrition:

*“it shows me ways to make tasty looking healthy snacks and meals and gives me ideas for short workouts and new exercises to mix up my work out”* (Female, 21).

Participants reported that they found advice to be practical and transferrable to their own lifestyle, as they could try exercises or recipes at home, or find information about upcoming fitness events. Another benefit mentioned was that access to advice and ideas on social media removed barriers such as having to pay for a gym membership or a personal trainer. For example, *“It also inspires me to do ‘at home’ workouts with minimal equipment rather than paying memberships/ attending personal training and classes.”* (Female, 21).

While many participants enjoyed sharing advice with each other, a minority of participants acknowledged the challenge of finding reliable and accurate information; they noted the unregulated nature of social media and potential for underqualified people to give inappropriate advice. These participants had a cynicism towards people posting in the fitspiration trend, saying many are *“uneducated and underqualified to give the advice they do”* (Female, 26). Some participants explicitly reported being careful about whose advice to follow; they specifically chose to follow people with relevant qualifications to ensure that the information was scientifically reliable and could be trusted. For example, one participant explained that *“the people i* [sic] *choose to follow have qualifications in the field … And base their methodology on science and evidence rather than on looks and their ability to market themselves or products.”* (Male, 23).

## Discussion

This study aimed to determine which types of fitspiration content people choose to access and how they engage with this content. Of interest, our results show that participants commonly accessed fitspiration content that has not been previously researched in great depth such as content related to ‘clean eating’, and social media posts generated by athletes and personal trainers. This supports the need for further research to examine the potential health outcomes of engaging with these types of content. For example, the notion of ‘clean eating’ has the potential to lead to the restrictive eating practices characteristic of orthorexia nervosa [37]. Also interesting was that the majority of participants passively viewed or liked content, but did not actively contribute fitspiration content themselves. It would be important to factor this knowledge in when recruiting for future fitspiration studies as a previous study recruited people who posted fitspiration on Instagram [17]. Furthermore, passive Facebook use has previously been associated with lower subjective wellbeing compared to active use [38].

We also sought to examine the health and wellbeing in our sample who engage with fitspiration. Of concern, 43% of our sample had high or very high levels of psychological distress, which is considerably higher than estimates in the general Australian population of 20% of females and 11% of males aged 18–24 years [39]. This finding generally aligns with those of a previous experimental study, which found that brief exposure to fitspiration leads to lower self-reported mood states [4]. The current study extends this previous finding to a real-word sample that choose to access fitspiration content, supporting an association between fitspiration usage and psychological distress that warrants further investigation. The proportion of the sample at risk of an eating disorder was also high at 17.7%. This proportion is similar to that of Holland and Tiggemann [17], who found that 17.5% of female participants who posted fitspiration on Instagram were at risk of an eating disorder, significantly greater compared to participants in their study who posted content about travel. While the current study generally supports this finding, the authors used a different measure to determine those at risk of an eating disorder, limiting our ability to directly compare the proportions between these two studies. The high proportion of our sample at risk of an eating disorder also further supports the finding from a previous study that found individuals who liked or followed fitspiration-related content were more likely to self-report having an eating disorder and misusing diet pills [18]. A greater proportion of participants in this study also had scores indicative of compulsive exercise behaviours (10%) compared to other studies using the EAI, which estimated risk for exercise addiction among males and females who regularly exercised at 3–7% [27, 40, 41]. Consistent with the findings of the present study, Holland and Tiggemann [17] found that people who posted in the fitspiration trend had higher scores on the Obligatory Exercise Questionnaire compared to those who posted about travel. Given this is a cross-sectional study, we are unable to determine whether the high proportion of psychological distress and disordered eating and exercise behaviours was caused by accessing fitspiration content. It is also possible that individuals with existing psychological distress and disordered eating and exercise behaviours may be predisposed to seek out fitspiration content. While several experimental studies support an immediate short-term effect of fitspiration on mood and body dissatisfaction [4, 13, 14], further research is required to explore the relationship between fitspiration and outcomes for health and wellbeing in the longer-term.

The majority of participants in this study reported positive benefits, including motivation to exercise and eat healthily, access to exercise ideas and being part of an online community. By providing motivation and social support fitspiration may lead to increased physical activity, which has significant benefits for physical and mental health [42, 43]. However, a minority of participants reported having experienced negative impacts which were often related to feeling inadequate or failing to achieve their goals. Furthermore, psychological distress and risk of an eating disorder or compulsive exercise behaviours were common in our sample. The presence of both positive and negative impacts suggests that fitspiration content may differentially influence individuals or that some pages and content are more harmful than others. Additionally, the frequency that individuals access fitspiration content may influence their perceptions and may mediate the potential impact on their health and wellbeing. Future research is needed to determine the individual and content-related factors associated with these negative and positive fitspiration experiences.

The benefits of social connection and interaction offered by the online fitspiration community can help to explain how fitspiration may enable active behaviour change. This concept is supported by previous studies, which have found that social support may enhance the efficacy of online interventions to improve physical activity and nutrition [44, 45]. However, through a sociological lens, the notion of this supportive community can also be reinterpreted as a social mechanism for regulating adherence to the ‘healthy practices’ and pursuit of an unattainable ideal currently endorsed by the fitspiration community [5]. Further research is required to examine whether fitspiration can genuinely contribute to balanced, attainable and sustainable behaviour change.

Despite outwardly reporting the benefits of fitspiration, the language that participants used and its underlying meaning revealed concerning findings embedded within their beliefs. Participants did not appear to realise that they had conflated their appearance ideal with optimal health, but justify their efforts to *look* fit with the belief that they are improving their overall health. Likewise, experimental research suggests that focussing on the functional aspects of idealised images actually has a negative effect on the appearance satisfaction of young female participants [46]. Fitspiration may be contributing to the construction and reinforcement of an ideal, attractive body type that is also perceived to be healthy, and also providing misinformation about what it is to look and be healthy with potentially harmful consequences. This aligns with previous studies that have found fitspiration posts often depict an appearance ideal that is lean, muscular and athletic [5, 8, 9]. Similar to Jong and Drummond [5], our results suggest that many users are internalising a particular appearance, and perceiving it as the ideal appearance of a healthy individual.

Open-ended responses from participants also offer some explanation for why content generated ‘everyday’ individuals was popular among our sample. Content generated by ‘everyday’ individuals was considered more relatable and trustworthy compared to celebrities or models. Similarly, a content analysis of fitspiration on Instagram found that personal accounts were more popular than commercial accounts, as indicated by a higher number of likes and followers [47]. In contrast to the findings of Jong and Drummond [5], who found that their participants (of a similar age, and also Australian) displayed less discernment of credibility and vested interests in their fitspiration role models, some participants within our sample demonstrated additional critical awareness through selecting more ‘realistic’ role models and acknowledging that goals may differ between individuals. However, it is not clear whether this content actually contains more reliable and beneficial information. Content may actually portray an idealised version of the ‘everyday person’, and therefore individuals may be comparing themselves to a glorified version of ‘normal’. Particularly as social media enables users to control how they present themselves to their social network by selectively posting content that reflects their desired image [3]. Furthermore, comparing one’s appearance to peers on social media has been shown to have a greater indirect effect on body image concerns than comparing to celebrities [48]. Future research is needed to determine whether fitspiration content created by ‘everyday’ individuals and personal trainers is reliable and beneficial given that these types of content are perceived as trustworthy and therefore have a greater potential to influence health beliefs and behaviours.

The present study has some limitations that are likely to have influenced the sample’s representativeness and our ability to confidently determine the relationship between health outcomes and engagement with fitspiration. This study used convenience sampling, in particular the majority of participants were recruited via Facebook and very few via Instagram, which is known to be a common source of fitspiration content [6]. We may have also recruited participants who are likely to spend more time on social media than those in the wider population who access fitspiration content [49]. Alternatively, reaching individuals who spend more time on social media may capture the audience most ‘at-risk’ and with higher exposure to fitspiration. The study had a low response rate; although, this is consistent with other studies that used social media recruitment for online surveys [17]. The amount of missing data (Table 2) suggests the survey may have been too lengthy, difficult or sensitive for some participants. Due to the study design, we were unable collect in-depth qualitative data about participants’ perception or to probe participants’ responses further. In particular, fewer responses from male participants meant their experiences, and the differences between female participants’ experiences, were unable to be explored in greater depth. However, this study can inform future qualitative research to capture a more comprehensive story of the influence of fitspiration. The questionnaire also included questions, particularly those relating to fitspiration content and engagement, that were generated by the researchers and these questions were not validated nor has their reliability been assessed. Finally, this study did not differentiate between different types of fitspiration content or characteristics of participants and therefore we were unable to determine how perceived harms and benefits may be attributed to particular types of content or individual characteristics. Comparing to a control group who do not engage with fitspiration would also be useful to determine the association between fitspiration and other health or demographic characteristics.

## Conclusion

Social media is an increasingly popular and accessible way to gather and share health-related information. This study has described the types of fitspiration content that users engage with and offers direction for future research into the potential impact of lesser-researched but popular types of content. Findings indicate that future research should broaden its scope to consider these other types of fitspiration-related content and their potentially associated harms with larger and more representative samples. This study also increases understanding in an emerging area of research about the potential negative impact of fitspiration on individuals who engage with this content, highlighted by the high prevalence of risk for compulsive exercise and eating behaviours and psychological distress in our sample. Fitspiration content may also be contributing unreliable health information and endorsing unrealistic appearance-related goals. While many participants report positive benefits associated with fitspiration, including social support and increasing motivation. Future and more in-depth research is needed to determine the individual risk factors as well as the types and features of content that are associated with either negative or positive fitspiration experiences.

## Abbreviations

BMI: Body Mass Index
EAI: Exercise Addition Inventory
EAT-26: Eating Attitudes Test-26 scale
IQR: Interquartile range
K10: Kessler 10 Psychological Distress Scale
